# FTO-Mediated m6A Demethylation of SERPINF1 Attenuates Multiple Myeloma Progression via the Wnt/β-Catenin Pathway

**DOI:** 10.4014/jmb.2510.10039

**Published:** 2026-02-11

**Authors:** Xiushuai Dong, Xi Chen, Yaoyao Tian, Lianjie Wang, Wei Wang

**Affiliations:** Department of Hematology, The Second Affiliated Hospital, Harbin Medical University, Harbin 150001, Heilongjiang, P.R. China

**Keywords:** Multiple myeloma, m6A demethylation, SERPINF1, FTO, Wnt/β-catenin

## Abstract

Multiple myeloma (MM) is an intractable hematologic malignancy characterized by clonal growth of malignant plasma cells in the bone marrow. Recent studies have highlighted the role of N6-methyladenosine (m6A) RNA modifications in MM progression; however, the function of the m6A demethylase fat mass and obesity–associated protein (FTO) remains unclear. This study aims to explore the mechanisms by which FTO-mediated m6A demethylation of Serpin Family F Member 1 (SERPINF1) impacts MM progression. SERPINF1 and FTO expressions were assessed via real-time quantitative polymerase chain reaction (RT-qPCR). The impact of such expressions on MM was evaluated using CCK-8, EdU, transwell, and tumor xenograft model assays. Key molecules involved in the Wnt/β-catenin pathway were assessed via Western blotting. The relationship between SERPINF1 and FTO was determined through correlation analysis, methylated RNA immunoprecipitation, luciferase, RT-qPCR, Western blotting, RNA immunoprecipitation, and actinomycin D treatment assays. Finally, the effect of their interaction on MM was assessed through rescue experiments. SERPINF1 expression was reduced in MM samples. SERPINF1 overexpression suppressed the malignant traits of MM cells and reduced the levels of β-catenin, c-Myc, and cyclin D1. *In vivo* experiments revealed that SERPINF1 overexpression suppressed tumor growth in xenograft models. Mechanistically, FTO expression was upregulated in MM and SERPINF1 expression was negatively regulated by demethylating its m6A sites via IGF2BP1. Rescue experiments demonstrated that SERPINF1 overexpression reversed FTO-induced oncogenic phenotypes. These findings suggest that FTO-mediated m6A demethylation suppressed SERPINF1 expression in MM, whereas SERPINF1 overexpression inhibited tumor progression via the Wnt/β-catenin pathway.

## Introduction

Multiple myeloma (MM) is a disorder of malignant plasma cells characterized by the clonal expansion of abnormal plasma cells within the bone marrow, which induces bone destruction, immunodeficiency, anemia, hypercalcemia, and renal dysfunction [1, 2]. It ranks second in prevalence among all hematological malignancies, accounting for nearly 10% of all blood cancers [3]. Despite substantial advances in therapeutic strategies, particularly with the emergence of proteasome inhibitors, monoclonal antibodies, immunomodulatory drugs, and autologous stem cell transplantation, MM remains largely untreatable for a substantial proportion of patients owing to inevitable relapse and development of drug resistance [4]. Hence, understanding the molecular pathogenesis of MM is critical for development of novel therapeutic targets and improving patient outcomes.

Serpin Family F Member 1 (SERPINF1), also called PEDF, is a secreted glycoprotein and member of the serpin family that lacks protease inhibitory activity but displays various biological effects such as anti-angiogenic, anti-inflammatory, and antiproliferative functions [5, 6]. One study found that SERPINF1 expression was upregulated in during glioma cell tumorigenesis to enhance glioma progression and stemness [7]. However, another study reported that metformin administration inhibited prostate cancer cell malignancy by upregulating the expression of SERPINF1, suggesting the inhibitory effects of SERPINF1 on prostate cancer progression [8]. Consequently, SERPINF1 may play different roles across cancer types. Unfortunately, the precise functional role of SERPINF1 in MM remains to be thoroughly investigated.

Recent studies have emphasized the importance of epigenetic and post-transcriptional modifications that regulate tumor behavior and contribute to therapeutic resistance in MM [9, 10]. Among these, N6-methyladenosine (m6A) RNA methylation has emerged as a key epitranscriptomic modification that influences RNA metabolism, encompassing stability, splicing, nuclear export, and translation [11]. It is the most ubiquitous internal modification in eukaryotic mRNAs and is dynamically regulated by enzymes such as methyltransferases (“writers”), demethylases (“erasers”), and binding proteins (“readers”) [12]. Several previous studies have focused on the roles of m6A methyltransferases and reader proteins such as METTL3, KIAA1429, ZC3H13, WTAP, YTHDF2, and HNRNPA2B1, whereas the role of m6A demethylases has received relatively little attention [13-16]. Among the known demethylases, fat mass and obesity–associated protein (FTO) and ALKBH5 have been found to regulate RNA metabolism and gene expression in some cancers [17, 18]. FTO, an obesity-related gene, functions as a prominent m6A demethylase [19]. By removing the methyl groups from adenosine residues, FTO modulates its target transcripts, thereby promoting the progression of different cancers such as acute myeloid leukemia, hepatocellular carcinoma, glioblastoma, and breast cancer [20]. One study demonstrated that FTO upregulation decreases m6A methylation in MM, promoting tumor growth and metastasis through the HSF1/HSP pathway in a YTHDF2-dependent manner [21]. Another research revealed that FTO drives bortezomib resistance by destabilizing SOD2 and facilitates MM progression via the HSF1/HSP axis [22]. These findings underscore the significance of FTO in the pathogenesis of MM and highlight the need for further exploration on its diverse molecular targets and mechanisms. Notably, no studies have investigated whether FTO regulates the expression of SERPINF1.

Therefore, the current study investigated how FTO-mediated m6A demethylation regulates SERPINF1 expression and contributes to MM progression. To further explore the underlying mechanisms, we examined the involvement of the Wnt/β-catenin pathway, focusing particularly on the expression of β-catenin. This study aimed to clarify the epitranscriptomic regulation of SERPINF1 by FTO and its potential role in modulating oncogenic signaling pathways in MM.

## Materials and Methods

### Clinical Samples

This study was approved by the ethics committee of our hospital (approval number: KY2022-102). Peripheral blood samples were obtained from 18 patients diagnosed with MM and from 18 age- and sex-matched healthy donors with normal physical examination results. All MM samples were obtained upon diagnosis prior to any therapeutic intervention. All peripheral blood samples were stored at −80°C. All patients provided written informed consent before participation. The clinical characteristics of the patients are outlined in Table 1.

### Cell Culture and Transfection

The three human MM cell lines, namely, U266B1 (CL-0510), AMO1 (CL-0687), and LP-1 (CL-1020), were purchased from Procell, China. U266B1 and AMO1 cells were cultured in RPMI-1640 medium (Procell) with 10% and 20% fetal bovine serum (FBS) (Procell), respectively, whereas LP-1 cells were maintained in IMDM medium (Procell) comprising 10% FBS. Human nucleus pulposus cells (HNPC, CP-H097Y, Procell) were cultured in HNPC complete culture medium (CM-H097Y, Procell). All cells were incubated under the conditions of 5% CO_2_ and 37°C.

SERPINF1, FTO, and IGF2BP1 overexpression plasmids (SERPINF1-OE, FTO-OE, and IGF2BP1-OE) were constructed by RiboBio (China) by employing the pcDNA3.1 vector, with an empty pcDNA3.1 vector as the negative control (NC-OE). For *in vitro* experiments, cells were transfected with the overexpression vectors utilizing Lipofectamine 3000 (Thermo Fisher Scientific, USA). For *in vivo* experiments, a lentiviral vector expressing SERPINF1, Lv-SERPINF1, was generated by cloning the SERPINF1 coding sequence into a lentiviral backbone. The recombinant lentivirus was packaged in U266B1 cells, and the viral supernatants were collected, concentrated, and administered into animal models.

### RNA Extraction and Real-Time Quantitative Polymerase Chain Reaction PCR (RT-qPCR)

The total RNA was extracted using TRIzol reagent (Invitrogen, USA). Thereafter, cDNA synthesis was performed using a reverse transcription kit (Takara, Japan). The generated cDNA was utilized to perform RT-qPCR with the help of SYBR Green Master Mix (Applied Biosystems, USA) and gene primers (Table 2), with GAPDH serving as an internal control. Data were quantitatively analyzed using the 2^−ΔΔCt^ method.

### Cell Counting Kit-8 (CCK-8) Assay

Transfected MM cells were seeded into 96-well plates (1 × 10^4^ cells/well) and incubated for 0, 24, 48, and 72 h. Upon the completion of each time point, CCK-8 reagent (10 μl, Beyotime, China) was added into each well, and cells were incubated for an additional 2 h at 37°C. The absorbance at 450 nm was detected by employing a microplate reader.

### EdU Assay

EdU incorporation was assessed using the Cell-Light EdU Apollo567 *in vitro* Kit (RiboBio, China) to evaluate DNA synthesis and determine MM cell proliferation. Briefly, transfected MM cells were seeded into 96-well plates (5,000 cells/well) and incubated for 24 h. Thereafter, the cells were treated with 10 μM EdU for 4 h, followed by fixation and permeabilization using 4% paraformaldehyde and 0.5% Triton X-100, respectively. After staining with Apollo567 for 30 min and counterstaining with DAPI, images were captured using a fluorescence microscope.

### Transwell Migration/Invasion Assays

Migration and invasion assays were performed using transwell inserts in 24-well plates. The migration assays used uncoated inserts, whereas in the invasion assays, the upper chamber was coated with 8% Matrigel (BD Biosciences, USA). The transfected MM cells were dispersed in 200 μl of serum-free media and added to the upper chamber, after which 600 μl of media containing 20% FBS was loaded into the lower chamber. After incubation for 24 h at 37°C, migrated or invaded cells through the membrane were fixed with 4% paraformaldehyde, stained with 0.1% crystal violet, and visualized using microscopy.

### Western Blotting

Proteins from transfected MM cells were extracted using RIPA buffer (Beyotime), and their concentrations were quantified using a bicinchoninic acid assay kit (Beyotime). Proteins were separated via sodium dodecyl sulfate–polyacrylamide gel electrophoresis and transferred onto polyvinylidene fluoride membranes. After blocking with 5% nonfat milk, membranes were incubated overnight at 4°C with the following primary antibodies: β-catenin antibody (A11512, ABclonal, China), c-Myc antibody (A1309, ABclonal), cyclin D1 antibody (A11022, ABclonal), SERPINF1 antibody (A3475, ABclonal), and GAPDH antibody (A19056, ABclonal). Thereafter, the membranes were exposed to HRP-conjugated goat anti-secondary antibodies (AS014, ABclonal) for 1 h at ambient temperature. Protein expression was then visualized using BeyoECL Plus chemiluminescent substrate (Beyotime).

### *In vivo* Xenograft Model

To investigate the role of SERPINF1 *in vivo*, nude mice (BALB/c; age, 4–6 weeks, male) purchased from Cyagen (China) were subcutaneously injected with U266B1 cells stably overexpressing SERPINF1 or the control vector. The tumor volume was evaluated every 5 days using the following formula: V = (length × width^2^)/2. Mice were then euthanized using pentobarbital sodium after 30 days, and tumors were excised, photographed, and weighed. The animal experiments were approved by the ethics committee of our hospital (approval number: 2023115).

### Methylated RNA Immunoprecipitation (Me-RIP) Assay

The MeRIP assay was performed using the Magna MeRIP m6A Kit (Millipore, USA) to examine m6A modifications on SERPINF1 transcripts. Total RNA was extracted from MM cells transfected with FTO-OE, FTO-OE + IGF2BP1-OE, or NC-OE vectors. RNA samples were chemically fragmented to approximately 100 nucleotides before immunoprecipitation. Fragmented RNA was exposed to protein A/G magnetic beads attached to either an anti-m6A or a control IgG antibody. Following immunoprecipitation, the enriched RNA was purified and analyzed via RT-qPCR to determine the relative m6A methylation levels on SERPINF1 mRNA.

### Luciferase Aassay

SERPINF1 wild-type vector (SERPINF1-WT) with m6A motifs and mutant SERPINF1 vector (SERPINF1-MUT) without m6A motifs were purchased from Obio Technology (China). SERPINF1-WT/SERPINF1-MUT were co-transfected with FTO-OE/NC-OE into MM cells. After 24-h incubation, relative luciferase activity was determined using the dual-luciferase reporter assay system (Promega, USA).

### Actinomycin D Treatment

After transfection, MM cells were treated 5 μg/ml actinomycin D (Sigma, USA) for 0, 3, and 6 h. At each time point, total RNA was isolated from MM cells to perform RT-qPCR. Finally, the SERPINF1 mRNA expression was calculated to reflect SERPINF1 mRNA stability.

### RNA Immunoprecipitation (RIP) Assay

RIP assay was performed to detect IGF2BP1 and SERPINF1 binding using the Magna RIP Kit (Millipore, USA). After lysing MM cells using RIP Lysis Buffer, the RNA-binding protein was immunoprecipitated using magnetic beads with anti-IGF2BP1 (A25715, ABclonal, China) and control IgG antibody. The co-precipitated RNAs were purified and analyzed using RT-qPCR to verify SERPINF1 enrichment.

### Statistical Analyses

All experiments were independently repeated three times, with each experiment including three biological replicates per group, resulting in nine biological replicates for each condition. Statistical analyses were conducted using GraphPad Prism 8.0. Data were presented as mean ± SD, and statistical significance was evaluated using two-tailed Student’s *t*-test or one-way analysis of variance (ANOVA) followed by Dunnett’s or Tukey’s *post hoc* test, with *p* < 0.05 indicating statistical significance.

## Results

### SERPINF1 Was Identified as a Key Downregulated Gene in MM and associated with the Wnt/β-Catenin Pathway

**Fig. 1:**
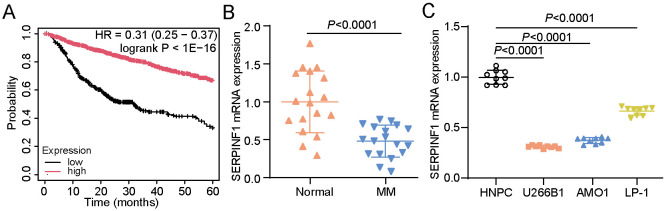
SERPINF1 is downregulated in multiple myeloma (MM). (**A**) Correlation between SERPINF1 expression and overall survival in MM analyzed using the public Kaplan–Meier plotter database. (**B**) SERPINF1 mRNA expression in clinical peripheral blood samples from 18 MM patients and 18 healthy donors analyzed via real-time quantitative polymerase chain reaction (RT-qPCR). Student’s *t*-test. (**C**) SERPINF1 mRNA expression measured in MM cell lines (U266B1, AMO1, and LP-1) and normal HNPC cells via RT-qPCR. Analysis of variance followed by Dunnett’s *post hoc* test. All data are presented as mean ± standard deviation from three independent experiments, each performed with three biological replicates (*n* = 9).

Kaplan–Meier plotter analysis (https://kmplot.com/analysis/index.php?p=home) revealed that low SERPINF1 expression was correlated with poor overall survival in MM (Fig. 1A). To validate this finding, RT-qPCR was performed on MM clinical and control samples. The results elucidated that SERPINF1 expression was notably lower in MM samples than in control samples (Fig. 1B). Further RT-qPCR analysis across human MM cell lines (U266B1, AMO1, and LP-1) and normal HNPC confirmed the downregulation of SERPINF1 expression in MM cell lines (Fig. 1C). Notably, analysis of our collected MM clinical samples revealed that β-catenin, c-Myc, and cyclin D1 expressions were significantly upregulated in MM samples (*P* < 0.0001, Table S1) and were strongly negatively correlated with SERPINF1 expression (Fig. S1). These findings indicate that SERPINF1 may be involved in MM progression and potentially modulate Wnt/β-catenin pathway.

### Overexpression of SERPINF1 Suppressed Malignant Features in MM Cells via the Wnt/β-Catenin Pathway

**Fig. 2:**
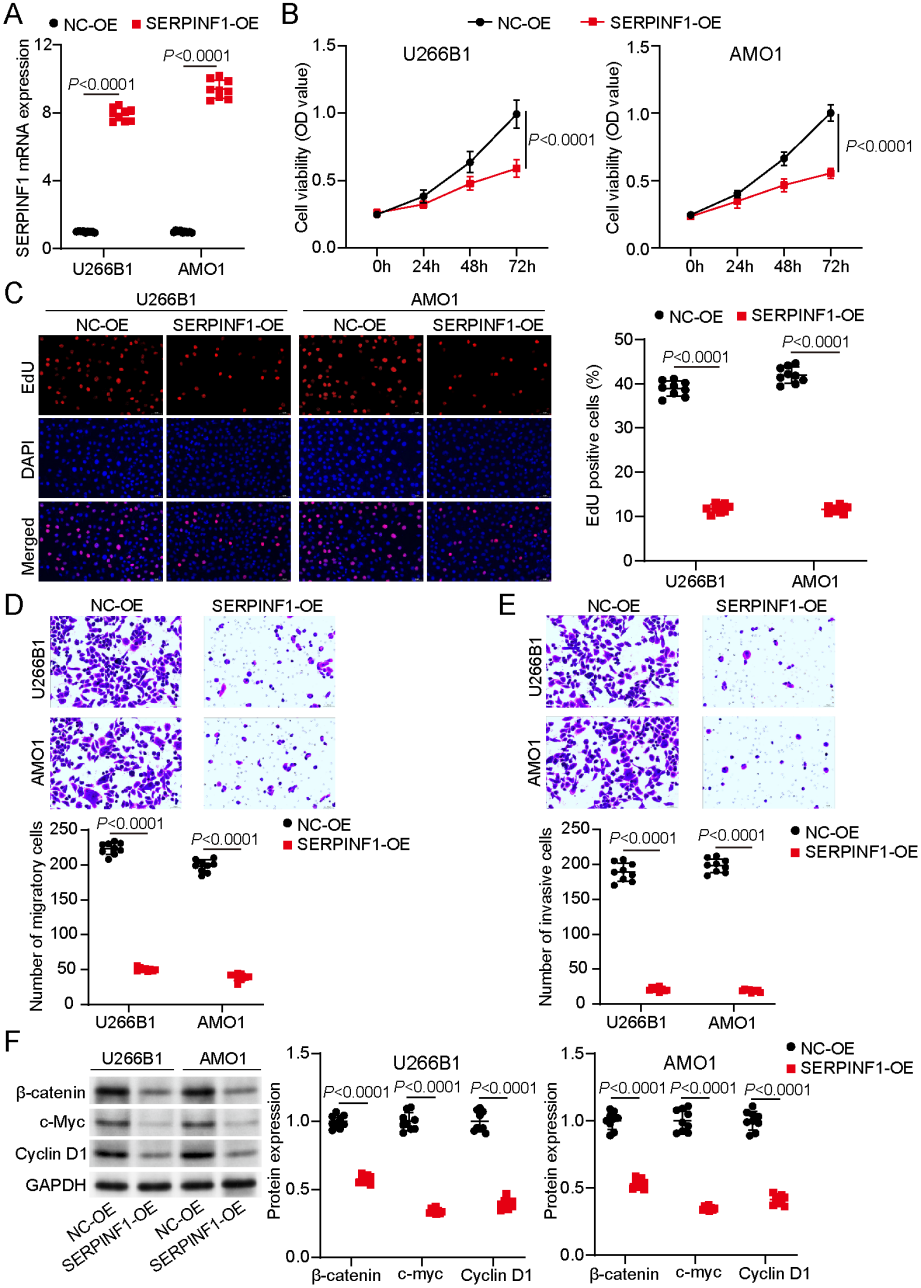
SERPINF1 overexpression suppressed multiple myeloma (MM) cell proliferation, migration, and invasion via the Wnt/β-catenin pathway. (**A**) SERPINF1 overexpression in MM cells validated via real-time quantitative polymerase chain reaction (RT-qPCR) following transfection. (**B**) MM cell viability after SERPINF1 overexpression assessed using the CCK-8 assay. (**C**) DNA synthesis of MM cells evaluated using the EdU assay after SERPINF1 overexpression. (**D**) Migratory rate of MM cells after SERPINF1 overexpression evaluated using the transwell migration assay. (**E**) Invasive capacity of MM cells following SERPINF1 overexpression assessed using the transwell invasion assay. (**F**) β-catenin, c-Myc, and cyclin D1 protein expression levels analyzed and quantified using Western blotting. Student’s *t*-test. All data are presented as mean ± standard deviation from three independent experiments, each performed with three biological replicates (*n* = 9).

The functional role of SERPINF1 in MM was explored by overexpressing SERPINF1 in U266B1 and AMO1 cell lines. These two cell lines were selected given that they demonstrated the most pronounced downregulation among the three MM cell lines analyzed (Fig. 1). The RT-qPCR results used to evaluate the transfection efficiency clearly indicated successful overexpression of SERPINF1 in both MM cell lines (Fig. 2A). The CCK-8 assay revealed that SERPINF1 overexpression markedly inhibited cell viability compared to NC cells (Fig. 2B). These findings were corroborated by those of the EdU incorporation assay, which revealed reduced DNA synthesis (Fig. 2C). Moreover, the transwell assays demonstrated that SERPINF1 overexpression markedly suppressed MM cell migration and invasion (Fig. 2D and 2E). Western blot analysis indicated a marked downregulation of β-catenin, c-Myc, and cyclin D1 expressions in SERPINF1-overexpressing cells (Fig. 2F). Collectively, these findings illustrate that SERPINF1 impedes cell proliferation, migration, and invasion and downregulates the Wnt/β-catenin pathway, thus functioning as a tumor suppressor.

### SERPINF1 Overexpression Inhibited Tumor Growth in a Xenograft Model via the Wnt/β-Catenin Pathway

**Fig. 3:**
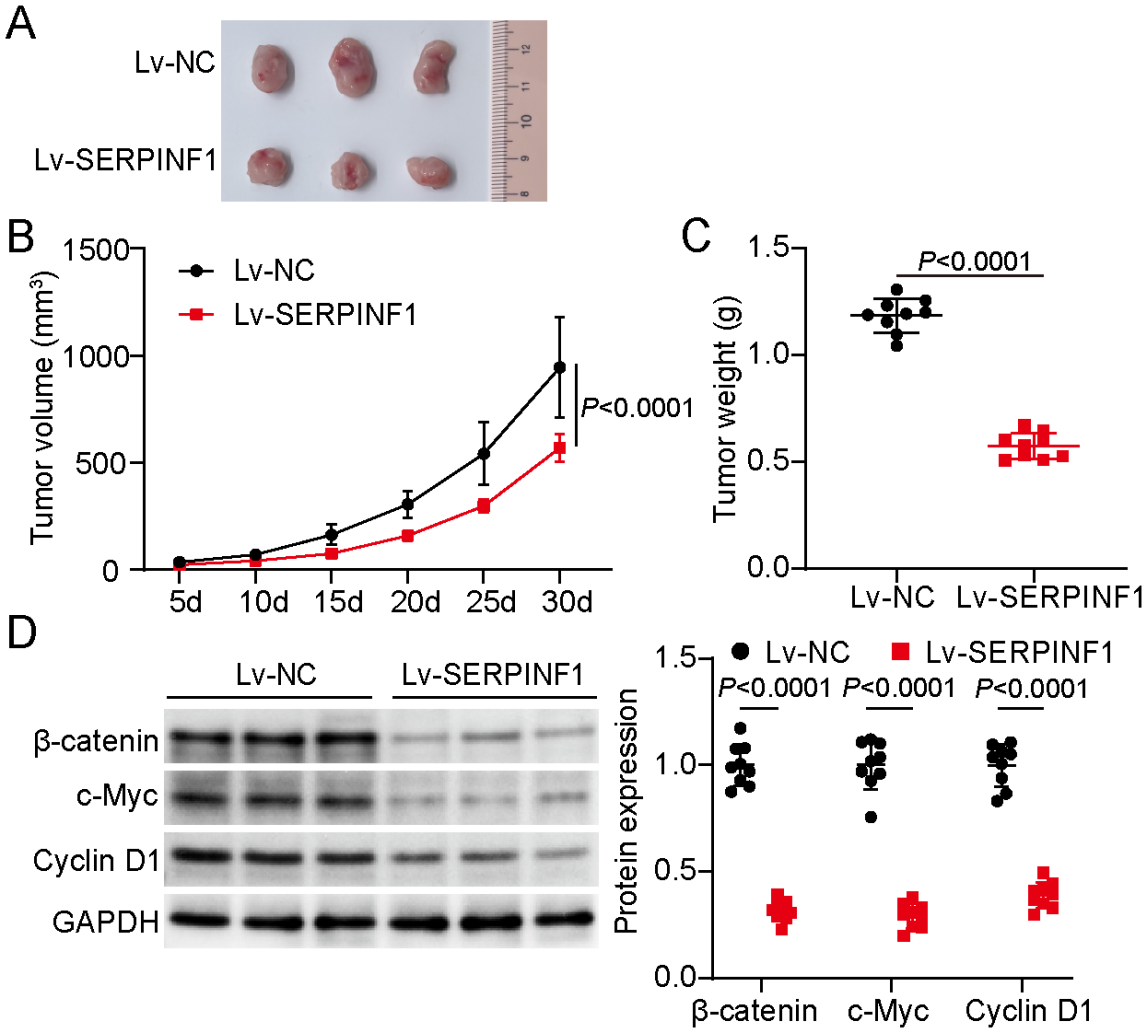
SERPINF1 overexpression inhibited tumor growth in a nude mouse xenograft model via the Wnt/β-catenin pathway. (**A**) Representative tumor images from different groups 30 days after injecting U266B1 cells with stable SERPINF1 overexpression or control. (**B**) Graph illustrating the growth of xenograft tumor volumes over time in the SERPINF1-overexpressing and control groups. (**C**) Tumor weights measured 30 days after injecting U266B1 cells with stable SERPINF1 overexpression or control. (**D**) β-Catenin, c-Myc, and cyclin D1 protein expression levels analyzed and quantified using Western blotting. Student’s *t*-test. Data are presented as mean ± standard deviation (SD) from three independent experiments, each performed with three biological replicates (*n* = 9).

To validate the tumor-suppressive effects of SERPINF1 *in vivo*, we established a xenograft model using U266B1 cells stably overexpressing SERPINF1 (see the Methods section). The tumors harvested after 30 days were clearly smaller following SERPINF1 overexpression (Fig. 3A). A marked reduction in tumor volume was observed over the course of the experiment, becoming particularly evident by day 30 (Fig. 3B). Consistent with these findings, tumor weights were also markedly reduced in the SERPINF1-overexpression group (Fig. 3C). Moreover, the protein levels of β-catenin, c-Myc, and cyclin D1 decreased in the SERPINF1-overexpression group (Fig. 3D). Collectively, these *in vivo* results further support the tumor-suppressive function of SERPINF1 in MM, particularly in its ability to limit tumor growth by inhibiting the Wnt/β-catenin pathway.

### FTO Negatively Regulated SERPINF1 Expression via IGF2BP1-Dependent m6A Modification in MM

**Fig. 4:**
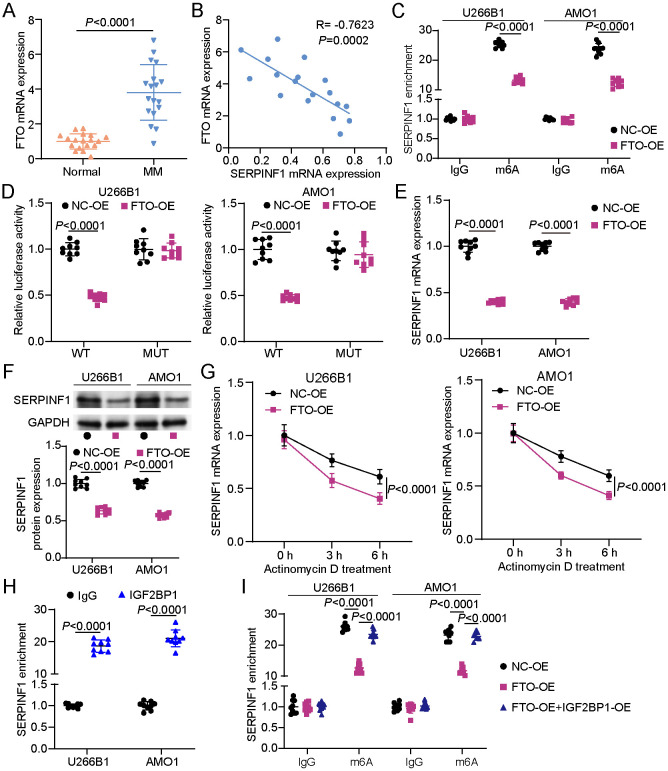
Fat mass and obesity–associated protein (FTO) negatively regulated SERPINF1 expression via m6A demethylation. (**A**) FTO mRNA expression in clinical peripheral blood samples from 18 MM patients and 18 healthy donors determined via real-time quantitative polymerase chain reaction (RT-qPCR). Student’s *t*-test. (**B**) Correlation between FTO expression and SERPINF1 in MM samples assessed via Pearson correlation analysis. (**C**) Relative m6A enrichment of SERPINF1 determined via the MeRIP assay following FTO overexpression vector (FTO-OE) transfection. Analysis of variance (ANOVA) followed by Tukey's *post hoc* test. (**D**) Relative luciferase activity of SERPINF1 wild-type (WT) and SERPINF1-mutant (MUT) measured in MM cells following FTO-OE transfection via the luciferase assay. ANOVA followed by Tukey’s *post hoc* test. (**E**) SERPINF1 mRNA expression determined in MM cells following FTO-OE transfection via RT-qPCR. Student’s *t*-test. (**F**) SERPINF1 protein expression in MM cells after FTO-OE transfection evaluated through Western blotting. Student’s *t*-test. (**G**) SERPINF1 mRNA stability in MM cells after FTO-OE transfection assessed via actinomycin D treatment. Student’s *t*-test. (**H**) Interaction between IGF2BP1 and SERPINF1 mRNA in MM cells verified via RIP assay. Student’s *t*-test. (**I**) SERPINF1 mRNA stability in MM cell lines after FTO-OE or FTO-OE + IGF2BP1-OE transfection assessed via actinomycin D treatment. ANOVA followed by Tukey’s *post hoc* test. All data are presented as mean ± standard deviation from three independent experiments, each performed with three biological replicates (*n* = 9).

To explore the role of m6A demethylase FTO in modulating SERPINF1 expression in MM, its expression levels were initially assessed. RT-qPCR analyses revealed that FTO expression was substantially upregulated in MM samples compared to controls (Fig. 4A). Correlation analysis of MM patient samples revealed a notable inverse relationship between FTO and SERPINF1 expression levels, suggesting a potential regulatory link (Fig. 4B). The MeRIP assay showed that FTO overexpression markedly decreased after m6A enrichment of SERPINF1 mRNA (Fig. 4C). The luciferase assay found that FTO overexpression markedly reduced the luciferase activity of the SERPINF1-WT group; however, it had no marked effect on the SERPINF1-MUT group (Fig. 4D). Furthermore, RT-qPCR analysis demonstrated that FTO overexpression markedly decreased SERPINF1 mRNA levels in MM cells (Fig. 4E). Similarly, SERPINF1 protein expression was also reduced following FTO overexpression (Fig. 4F). After actinomycin D treatment, FTO overexpression impaired the stability of SERPINF1 mRNA (Fig. 4G). Given that m6A-mediated mRNA is regulated by the m6A reader, we further explored the involvement of m6A readers in SERPINF1 regulation. RIP assay confirmed a direct interaction between IGF2BP1 and SERPINF1 in MM cells (Fig. 4H). Moreover, co-transfection with IGF2BP1 overexpression vector reversed the decrease in m6A enrichment of SERPINF1 mRNA caused by FTO overexpression (Fig. 4I). These results indicate that FTO negatively regulates SERPINF1 expression by reducing IGF2BP1-dependent m6A modification.

### SERPINF1 Overexpression Partially Reversed the FTO-Mediated Oncogenic Phenotypes in MM Cells via the Wnt/β-Catenin Pathway

**Fig. 5:**
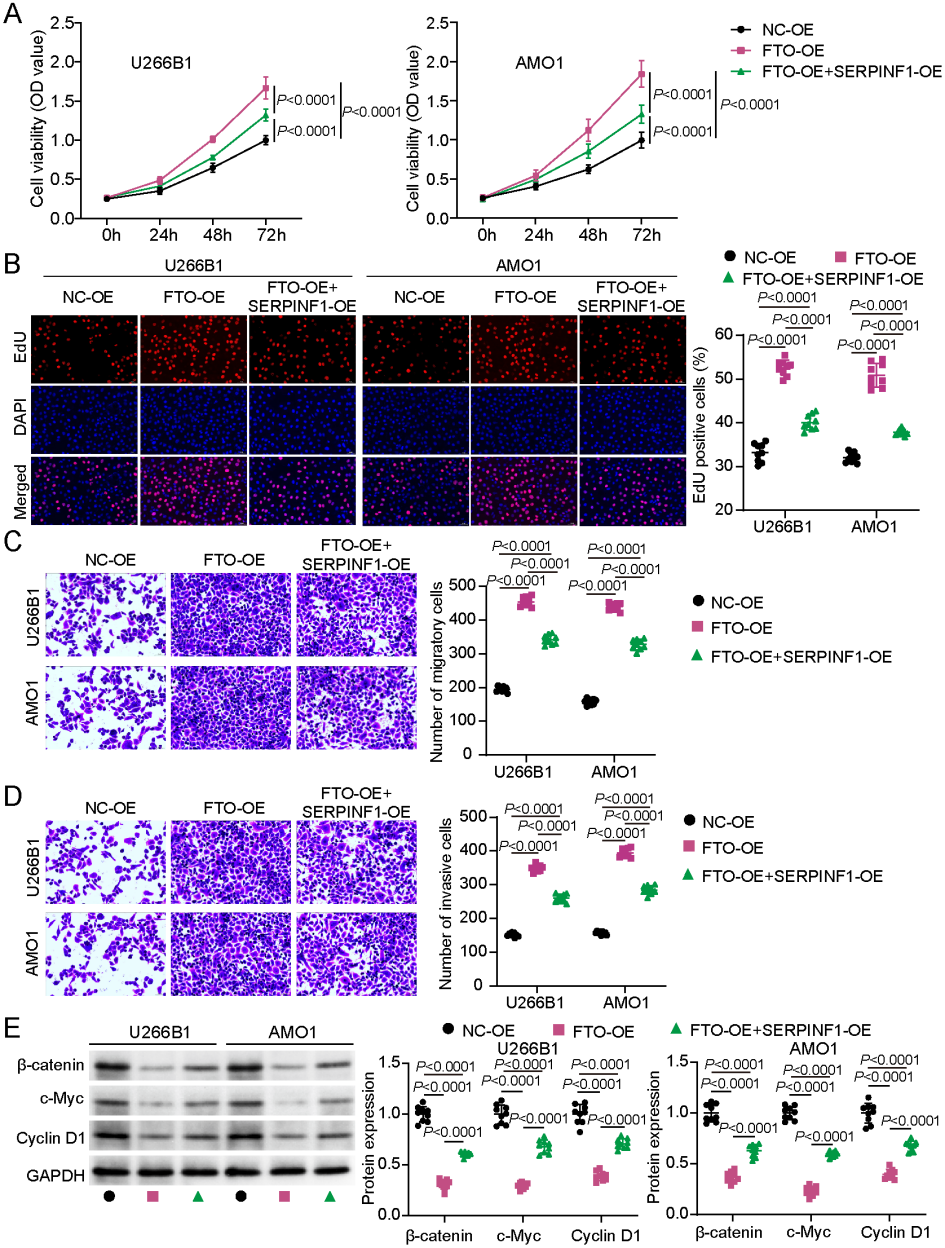
SERPINF1 overexpression partially reversed fat mass and obesity–associated protein (FTO)–mediated oncogenic effects in MM cells via the Wnt/β-catenin pathway. MM cell lines were transfected with NC-OE, FTO-OE, or FTO-OE vectors combined with SERPINF1-OE for rescue experiments. (**A**) Cell proliferation in the aforementioned transfected MM cells assessed via the CCK-8 assay. (**B**) DNA synthesis in these cells assess via EdU assays. (**C**) Cell migration in the aforementioned transfected MM cells evaluated using the transwell migration assay. (**D**) Invasion of MM cells following the aforementioned transfections evaluated via the transwell invasion assay. (**E**) β-catenin, c-Myc, and cyclin D1 protein expression levels in MM cells following the aforementioned transfections analyzed and quantified via Western blotting. Analysis of variance followed by Tukey’s *post hoc* test. All data are presented as mean ± standard deviation from three independent experiments, each performed with three biological replicates (*n* = 9).

To further explore the functional interplay between FTO and SERPINF1, rescue experiments were conducted by overexpression of SERPINF1 in FTO-overexpressing MM cells. CCK-8 results showed that SERPINF1 overexpression negated the FTO-induced increase in cell viability (Fig. 5A). Similarly, the EdU assay revealed that SERPINF1 overexpression counteracted the FTO-mediated enhancement in DNA synthesis (Fig. 5B). SERPINF1 overexpression restored the inhibition of migration and invasion induced by FTO overexpression in MM cells (Fig. 5C and 5D). Additionally, SERPINF1 overexpression reversed the decrease in protein levels (β-catenin, c-Myc, and cyclin D1) caused by FTO overexpression in MM cells (Fig. 5E). Collectively, these findings suggest that FTO promotes MM progression by suppressing SERPINF1 expression via m6A demethylation. Restoring SERPINF1 expression antagonizes FTO-mediated oncogenic effects by regulating the Wnt/β-catenin pathway in MM.

## Discussion

Although advances in therapeutic approaches for MM have notably improved patient outcomes, the poor prognosis of patients with high-risk disease remains a major clinical challenge [23]. This persistent concern highlights the urgent need to deepen the current understanding regarding the underlying regulatory mechanisms driving MM, in hopes of identifying more effective targeted therapeutic strategies. In the current study, we elucidated a novel regulatory mechanism in MM involving FTO-mediated suppression of SERPINF1 and its downstream effect on the Wnt/β-catenin pathway. Through comprehensive *in vitro* and *in vivo* analyses, our study results showed that SERPINF1 contributes to tumor suppression in MM, whereas FTO acts as an oncogene by downregulating SERPINF1 expression through m6A demethylation. Our findings substantiate the FTO–SERPINF1–Wnt axis as a potential therapeutic target for the treatment of MM.

We performed a prognosis analysis identifying SERPINF1 expression was correlated with poor overall survival in MM. Notably, studies have shown that SERPINF1 functions as a cancer suppressor in several other malignancies, such as in pancreatic, breast, prostate, and lung cancers, suggesting its potential tumor-suppressive role in MM [24-27]. In the context of MM, wherein angiogenesis and metastatic spread are hallmark features of disease progression, SERPINF1 suppression may remove critical constraints on tumor growth and dissemination. Our current study revealed that SERPINF1 overexpression suppressed MM cell proliferation, migration, and invasion, corroborating its tumor-suppressive capabilities. This finding concurs with those of earlier work identifying SERPINF1 as a negative regulator of tumor progression, including suppression of tumor growth and metastatic behavior [6]. Our *in vivo* studies further substantiated the tumor-suppressive role of SERPINF1 using xenograft models. These findings are particularly relevant given the current lack of therapeutic approaches for MM and the need for therapies that can effectively control disease progression and improve patient survival. Furthermore, the results of the Western blot analysis revealed a substantial reduction in β-catenin protein levels upon SERPINF1 overexpression, suggesting that the Wnt/β-catenin pathway is a downstream target of SERPINF1.

The Wnt/β-catenin pathway has been widely recognized for its critical role in the pathogenesis of MM, particularly its influence on cell survival, stemness, and drug resistance [28, 29]. Aberrant activation of Wnt signaling aids in MM cell survival, proliferation, osteolysis, and apoptosis resistance [30]. β-Catenin, a central mediator of canonical Wnt signaling, translocates to the nucleus upon pathway activation and drives the expressions of numerous oncogenic genes, including cyclin D1 and c-MYC [31]. Given the essential role of the Wnt/β-catenin pathway in MM progression, regulatory mechanisms that influence this pathway are of considerable therapeutic interest. Our findings support the notion that restoring SERPINF1 expression can inhibit Wnt signaling and potentially reverse MM-associated phenotypes. Importantly, the interplay between SERPINF1 and β-catenin provides a mechanistic link that could be exploited for therapeutic benefit.

Despite available studies on the oncogenic role of FTO in multiple malignancies, the downstream effectors through which FTO drives MM progression remain incompletely defined. A key novel contribution of the present study is the identification of SERPINF1 as a previously unrecognized, functionally essential downstream target of FTO in MM, thereby extending the mechanistic framework of FTO-mediated tumorigenesis beyond its established targets. FTO, as an m6A eraser, has been shown to exert oncogenic effects in various malignancies. For instance, one study showed that FTO was elevated in hepatocellular carcinoma and facilitated tumor progression by stabilizing BUB1 mRNA by inducing m6A demethylation, consequently triggering the activation of TGF-β signaling [32]. Another study showed that FTO promoted chemoresistance in colorectal cancer by stabilizing NUPR1 mRNA via m6A demethylation [33]. In contrast, studies have also shown that FTO possesses tumor-suppressive functions in clear cell renal cell carcinoma and ovarian cancer [34, 35], underscoring the complexity and tissue specificity of FTO-mediated epitranscriptomic regulation. In acute myeloid leukemia, FTO acts as an oncogene by decreasing m6A levels on ASB2 and RARA mRNAs, thereby promoting leukemogenesis and impairing differentiation [36]. Similar to a previous study in acute myeloid leukemia, our study found that FTO was overexpressed in MM samples, suggesting its oncogenic role in MM. However, our study describes a specific FTO–SERPINF1 regulatory axis linking m6A demethylation to the suppression of a tumor-suppressive secreted factor. We demonstrated that SERPINF1 mRNA is directly modified by m6A and that FTO overexpression notably reduces its m6A enrichment, thereby decreasing mRNA stability and expression. Importantly, no previous study has identified SERPINF1 as an m6A-regulated target in MM or described its epitranscriptomic regulation by FTO. Our rescue experiments further verified that SERPINF1 overexpression attenuated the oncogenic phenotypes induced by FTO. These findings support a direct regulatory axis where FTO-mediated demethylation suppresses SERPINF1, thereby enhancing MM progression. The inverse correlation between FTO and SERPINF1 expression further substantiates the suppressive role of FTO on SERPINF1. Collectively, our results provide novel mechanistic insights that FTO drives MM progression, that is, by enhancing oncogenic signaling and by actively repressing a tumor-suppressive gene through m6A-dependent post-transcriptional regulation. This FTO–SERPINF1 axis, therefore, represents a distinct and previously unrecognized layer of epitranscriptomic control in MM, with potential implications for targeted therapeutic intervention.

This study has been the first to show that SERPINF1 expression, which is regulated by the m6A demethylase FTO, suppresses MM progression via the Wnt/β-catenin pathway. However, several limitations of the present study should be acknowledged. First, despite identifying SERPINF1 as a key target of FTO, other m6A-regulated genes and pathways may also contribute to disease progression, highlighting the need for additional investigations. Moreover, although the current findings were validated in cellular and animal models, larger preclinical models and clinical trials are required to confirm the therapeutic efficacy of FTO inhibitors and SERPINF1 restoration.

In conclusion, our study uncovered a novel epigenetic mechanism wherein FTO-mediated m6A demethylation of SERPINF1 drives MM progression through the Wnt/β-catenin pathway. Notably, our findings identified SERPINF1 as a key tumor suppressor whose reactivation may hinder disease advancement. These findings expand the current understanding of MM pathobiology and provide a foundation for future research aimed at translating these molecular findings into clinically applicable therapeutic strategies.

## Supplemental Materials

Supplementary data for this paper are available on-line only at http://jmb.or.kr.



## Figures and Tables

**Table 1:** Clinical characteristics of 18 multiple myeloma patients.

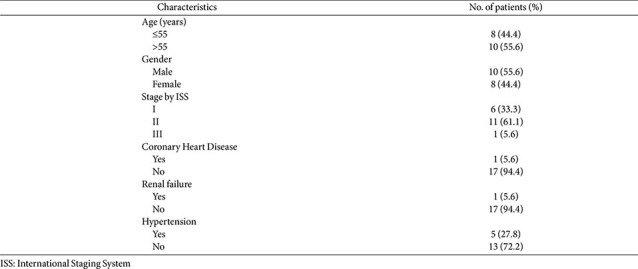

**Table 2:** Primer sequences used in qRT-PCR analysis.

Gene		Sequence (5'-3')
SERPINF1	Forward	TGAAGGCGAAGTCACCAAGTCC
	Reverse	CCATCCTCGTTCCACTCAAAGC
FTO	Forward	GACCTGTCCACCAGATTTTCA
	Reverse	AGCAGAGCAGCATACAACGTA
GAPDH	Forward	CCTGGTATGACAACGAATTTG
	Reverse	CAGTGAGGGTCTCTCTCTTCC
