# Supplementary material for: FTO-Mediated m6A Demethylation of SERPINF1 Attenuates Multiple Myeloma Progression via the Wnt/β-Catenin Pathway
Source: J Microbiol Biotechnol. 2026 Feb 11;36:e2510039. doi: 10.4014/jmb.2510.10039 (PMC12935505; doi:10.4014/jmb.2510.10039)
Supplement: Supplementary file 1 [file jmb-36-e2510039-supple.pdf]

J. Microbiol. Biotechnol. 2026. 36: e2510039

https://doi.org/10.4014/jmb.2510.10039

Supplementary Figure and Table

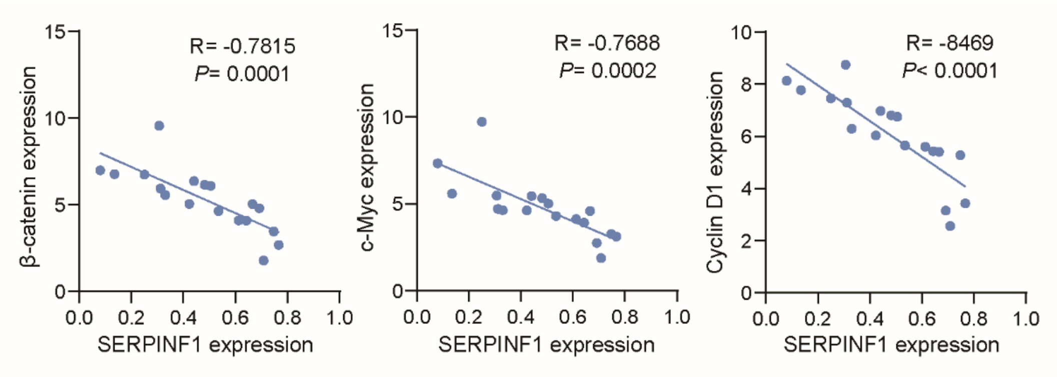

Fig. S1. The correlation between SERPINF1 expression and Wnt/β-catenin components in our collected 18 MM samples by Pearson correlation analysis.

Table S1. The expression pattern of canonical Wnt/β-catenin components within our collected clinical samples.

Gene Symbol	Fold change	*P* value
β-catenin	5.328	<0.0001
c-Myc	4.775	<0.0001
Cyclin D1	6.046	<0.0001
